# Improved Photocatalytic Activity via *n*-Type ZnO/*p*-Type NiO Heterojunctions

**DOI:** 10.3390/nano12203665

**Published:** 2022-10-18

**Authors:** Ligang Ma, Xiaoqian Ai, Yujie Chen, Pengpeng Liu, Chao Lin, Kehong Lu, Wenjun Jiang, Jiaen Wu, Xiang Song

**Affiliations:** 1 School of Electronic Engineering, Nanjing Xiaozhuang University, Nanjing 211171, China; 2 School of Physics and Information Engineering, Jiangsu Province Engineering Research Center of Basic Education Big Data Application, Jiangsu Second Normal University, Nanjing 210013, China

**Keywords:** ZnO/NiO heterojunctions, photocatalytic activity, microstructure

## Abstract

The design and construct *pn* heterojunction to reduce the recombination rate of photogenerated electron-hole pairs can effectively improve photocatalytic activity. In this study, ZnO/NiO heterojunctions were fabricated by annealing a Zn/Ni metal organic framework precursor synthesized via coprecipitation. The effects of the precursor annealing temperature on the microstructure, morphology, and optical properties of the ZnO/NiO nanocomposites were investigated using X-ray diffraction, scanning electron microscopy, transmission electron microscopy, X-ray photoelectron spectroscopy, and UV-vis absorption spectroscopy. The results showed that the nanocomposite was composed of hexagonal wurtzite ZnO and cubic NiO, with the former being the dominant phase. Large ZnO nanoparticles were attached to small NiO nanoparticles, and a *pn* heterojunction interface was formed. The photodegradation performance of the nanomaterials was evaluated by monitoring the degradation of RhB under irradiation by ultraviolet light. The ZnO/NiO nanocomposites exhibited excellent photocatalytic activity when the annealing temperature was 550 °C. The photodegradation mechanism was also analyzed in detail, revealing that the heterojunction between the *n*-type ZnO and the *p*-type NiO played an important role in impeding the recombination of photogenerated electron-hole pairs and improving the photocatalytic efficiency.

## 1. Introduction

Over the past decades, the development and use of traditional energy sources have resulted in significant benefits [1]. Although these energy sources have facilitated several benefits, they also cause harm. For example, oil is a fuel for cars and machines, but its use seriously pollutes the environment owing to the release of contaminants [2], including heavy metal [3] and organic pollutants [4]. In addition, the widespread presence of pharmaceutical materials in the environment has resulted in ecosystems being adversely affected, including human life [5]. Therefore, in recent years, new energy sources have been explored to replace traditional ones, and environmentally friendly means are being sought after [6]. For example, the development of technology that harnesses inexhaustible solar energy (e.g., solar cells) reduces the need to exploit traditional energy sources [7]. Furthermore, the development of materials with high catalytic activity can reduce the amount of heavy metals and organic pollution [8].

Semiconductor photocatalysts have been widely used to remove organic and inorganic contaminants or heavy metal ions from wastewater [8]. The photocatalysis mechanism involves irradiating the material with light of a wavelength higher than the band gap of the material to excite photogenerated electron-hole pairs. The latter are transported to the material surface and undergo redox reactions with oxygen and water in a dye solution [9]. The photogenerated electrons react with the oxygen dissolved in water to form superoxide radicals, and the holes react with water to form hydroxyl radicals. These two types of free radicals destroy organic dyes and transform them into clean products, such as carbon dioxide and water, which are beneficial to the environment. These degradation mechanisms have been widely discussed in the literature. Common catalyst semiconductors include CdS [10], ZnO [11,12], TiO_2_ [13], and CuO [14], among others. However, in single-phase materials, the photogenerated electron-holes pairs excited by irradiation can easily recombine, significantly affecting the photodegradation performance [15]. Therefore, the photocatalytic performance can be improved by the doping [16], modification, and loading of metal particles [17] on semiconductors, thereby improving the separation of photogenerated electron-hole pairs. 

Among many metal oxide semiconductors, ZnO nanomaterials (naturally formed as *n*-type semiconductors with a band gap of 3.4 eV) have excellent performance in photocatalytic processes because of their high photosensitivity, high photocatalytic activity, suitable bandgap, low cost, and environmental friendliness [18]. In addition, NiO [19,20,21] is a *p*-type semiconductor with a band gap of 3.5 eV [22], and it has a lattice mismatching degree with *n*-type ZnO, which is beneficial for the formation of *pn* heterojunctions [23]. This establishes a built-in electric field in the heterojunction to facilitate the separation of charges and improve photocatalytic activity. Therefore, it is very important to find low-cost, high-quality, and high-performance nano-heterojunctions between ZnO and NiO. Manal Hessien et al. [24] prepared ZnO–NiO nanocomposites using a combining Neem leave extract with hydrothermal synthesis. Nagabandi Jayababu et al. [25] synthesized ZnO–NiO nanocomposites using co-precipitation followed by sol-gel process. In addition, the coprecipitation method was used to synthesize ZnO–NiO nanocomposites with some surfactants [26]. Recently, nanostructures, such as metal oxides or metal sulfides, have been derived from a porous metal–organic framework with large surface areas by various annealing methods [27,28,29,30]. In this way, the organic framework structure is decomposed by annealing or treatment at high temperature under atmosphere, while the metal elements combine with atmosphere to form a metallic compound. At the same time, the metal compound also retains the porous framework structure under suitable conditions. Most of the literature reports that the mono-metal organic framework structure forms only one metal oxide [31,32]. 

In this study, a duplex porous metal–organic framework structure consisting of Zn and Ni was designed and synthesized by a simple and low-cost coprecipitation method, and then was annealed at different temperatures to form ZnO/NiO nanocomposites. X-ray diffraction (XRD) analysis, scanning electron microscopy (SEM), transmission electron microscopy (TEM), X-ray photoelectron spectroscopy (XPS), and UV-vis absorption spectroscopy were used to investigate the microstructure, morphology, and optical properties of the ZnO/NiO nanocomposites. Finally, the photocatalytic degradation performance of the nanocomposites was examined, and the degradation mechanism was analyzed in detail.

## 2. Experimental Method

### 2.1. Materials

Nickel nitrate hexahydrate (Ni(NO_3_)_2_·6H_2_O, 99.9%), methanol, 2-methylimidazole (98%) and zinc nitrate hexahydrate (Zn(NO_3_)_2_·6H_2_O, 99%) were purchased from Sinopharm Chemical Reagent Co., Ltd, Shanghai, China. All chemical reagents were of analytical grade and were used without further purification.

### 2.2. Experimental Process

**Scheme 1:**
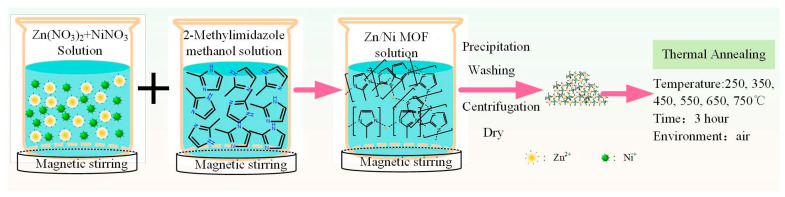
Schematic diagram of the synthesis process of ZnO/NiO nanocomposites.

The synthesis process of the ZnO/NiO nanocomposites is shown in Scheme 1. Specifically, Zn(NO_3_)_2_·6H_2_O (4.46 g) and Ni(NO_3_)_2_·6H_2_O (4.36 g) were added to methyl alcohol (250 mL), and the mixture was stirred at 80 °C for 60 min, which was labeled as Solution A. This solution was poured into Solution B, in which 2-dimethylimidazole (9.85 g) and methyl alcohol (250 mL) were mixed, and the mixture of Solution A and B was stirred at 80 °C for 60 min. Finally, the precipitate was separated by centrifugation at 7000 rpm and dried to obtain a Zn/Ni precursor (Zn/Ni-PR). To investigate the effect of the annealing temperature on the photodegradation performance, the sample was annealed in a tube-shaped sintering furnace, and the Zn/Ni-PR sample was annealed at different temperatures (250 °C, 350 °C, 450 °C, 550 °C, 650 °C, and 750 °C). The annealing time was set to 3 h and the heating rate was 2 °C/min in air. The samples were labeled as Zn/Ni-*x*, where *x* is the annealing temperature (e.g., the sample treated at 250 °C was labeled as Zn/Ni-250.

### 2.3. Characterization

The microstructures of the Zn/Ni-PR and Zn/Ni-*x* samples were investigated using a Rigaku Dmax-rB powder diffractometer with Cu Kα radiation (*λ* = 1.5418 Å) over the 2θ range of 20 to 80°, and the operating voltage and current were 40 kV and 60 mA, respectively. The morphology was characterized using a ZEISS Gemini 500 field-emission scanning electron microscope (FE-SEM). Fine and micro-connective forms were investigated using a high-resolution transmission electron microscope (HRTEM, FEITecnai G2 F30, USA), which was attached to an energy dispersive spectroscopy facility, at an accelerating voltage of 200 kV. For the TEM measurements, the powder sample was dispersed in ethanol via ultrasonication to obtain a suspension, and it was subsequently dropped onto a Cu grid. UV-vis absorption spectra were measured using a UH4150 instrument (Hitachi Corp., Tokyo, Japan) equipped with an integrating sphere. All measurements were performed at room temperature. The electrochemical impedance spectroscopy (EIS) was determined by CHI660E three-electrode electrochemical workstation at Shanghai Chenhua. The Pt foil was used as a counter electrode, the glassy carbon electrode as a working electrode, and Ag/AgCl as a reference electrode. A 0.5 M Na_2_SO_4_ solution was used as the electrolyte. The photocatalytic activities of Zn/Ni-PR and Zn/Ni-*x* were examined by evaluating the degradation of organic dyes under ultraviolet light. We selected RhB organic dyes as model pollutants. The photodegradation process was conducted as follows: (1) The photocatalyst (50 mg) was added to 50 mL of the organic dye (10 mg/L) by ultrasonic dispersion for 10 min; (2) To achieve a balance between adsorption and desorption, the catalyst containing the organic dye was isolated from any sources of light for 1 h and stirred continuously. No precious metal catalysts were added during the degradation process; (3) Prior to illumination, the reaction solution (3 mL) was extracted and centrifuged to obtain a clear supernatant; (4) A 365 nm light emitting diode (LED) with a power of 320 mW/cm^2^ was used to degrade the organic dye. The absorption spectrum of the clear supernatant solution was measured using a UH4150 instrument to characterize the degradation performance.

## 3. Results and Discussion

**Figure 1:**
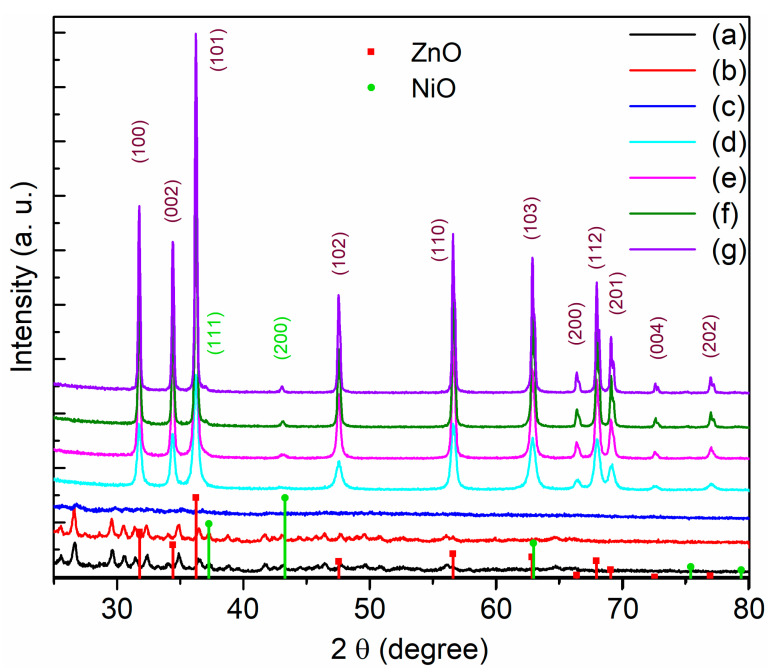
X-ray diffraction of the powder for (a) Zn/Ni-PR, (b) Zn/Ni-250, (c) Zn/Ni-350, (d) Zn/Ni-450, (e) Zn/Ni-550, (f) Zn/Ni-650, and (g) Zn/Ni-750.

Figure 1 shows the X-ray diffraction (XRD) patterns of the Zn/Ni-PR and Zn/Ni-*x* samples. The Zn/Ni-PR and Zn/Ni-250 samples had similar diffraction patterns, and the intensities of their diffraction peaks were weak. In addition, there were no diffraction peaks corresponding to ZnO and NiO. This is because the precursor did not form ZnO and NiO with oxygen in the air, and the organic framework was not completely decomposed at this temperature. When the annealing temperature was increased to 350 °C, the weak diffraction peaks disappeared, and the sample was transformed into the amorphous phase (i.e., the organic components in the sample were decomposed). When the annealing temperature was higher than 450 °C, obvious diffraction peaks appeared at 2θ = 31.7°, 34.4°, 36.2°, 47.5°, 56.7°, 62.9°, 66.4°, 68.0°, and 69.2°. According to JCPDS card No. 80-0075 [33], we confirmed that these diffraction peaks corresponded to (100), (002), (101), (102), (110), (103), (200), (112), and (201) crystal planes of ZnO with a hexagonal wurtzite structure. Weak diffraction peaks at 37.3° and 43.3°, which belonged to (111) and (200) crystal planes of cubic wurtzite NiO (JCPDS No. 47-1049) [34], were also detected (the locations are marked by green circles in the figure). Moreover, as the annealing temperature increased, the intensities of all diffraction peaks gradually increased. This occurred because the precursor reacted adequately with oxygen at high temperatures to form ZnO with a hexagonal wurtzite structure and NiO with a cubic structure, and the organic framework decomposed. According to the area enclosed by the diffraction peaks shown in Figure 1, the ZnO content was higher than that of NiO, and as a main component, ZnO played a major role in the ZnO/NiO nanocomposites.

**Figure 2:**
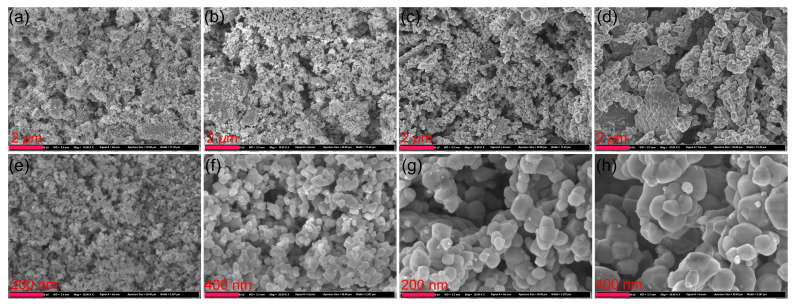
Top row: low-resolution SEM morphology of (**a**) Zn/Ni-450, (**b**) Zn/Ni-550, (**c**) Zn/Ni-650, and (**d**) Zn/Ni-750. Bottom row: high-resolution SEM morphology of (**e**) Zn/Ni-450, (**f**) Zn/Ni-550, (**g**) Zn/Ni-650, and (**h**) Zn/Ni-750.

**Figure 3:**
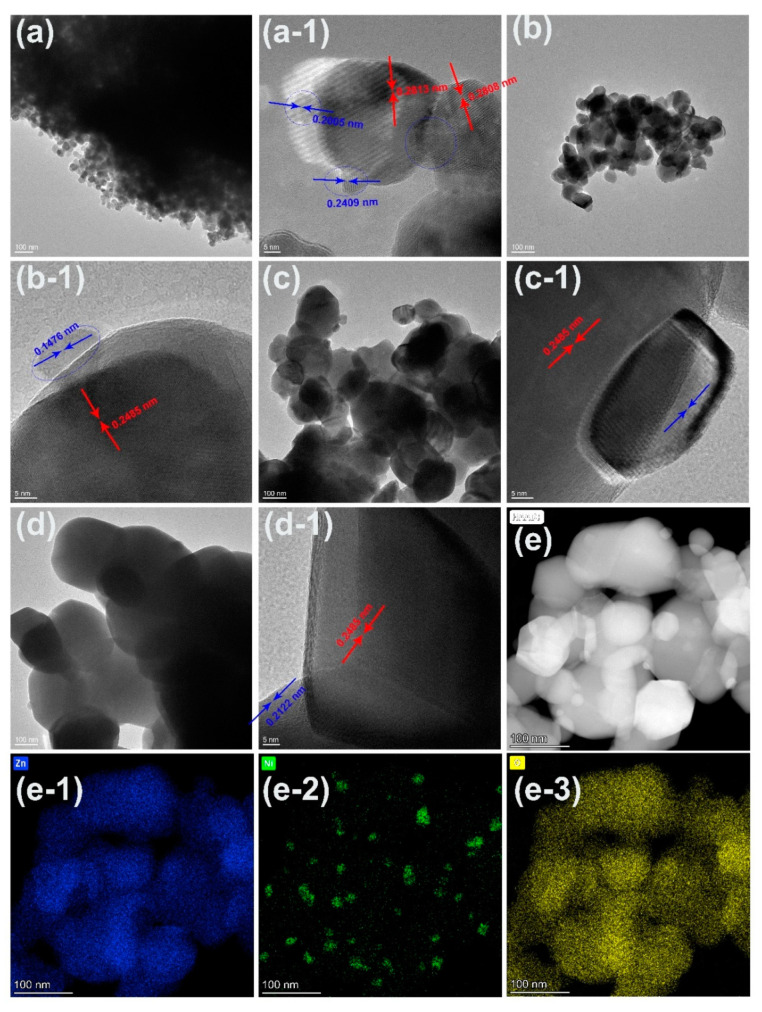
TEM images of Zn/Ni-PR annealed at (**a**) 450, (**b**) 550, (**c**) 650, and (**d**) 750 °C. Panels (**a-1**,**b-1**,**c-1**,**d-1**) display the high-resolution TEM images corresponding to panels (**a**–**d**), respectively. (**e**) High-angle annular dark field image morphology for the sample annealed at 550 °C. Panels (**e-1**–**e-3**) show the Zn, Ni, and O mapping of the image shown in (**e**), respectively.

Figure 2 shows low and high-resolution SEM images of the sample annealed at temperatures above 450 °C. The size of the nanoparticles increased gradually as the annealing temperature increased. The high-resolution SEM morphology images indicate that large nanoparticles were attached to small nanoparticles. The ZnO and NiO particles formed a heterogeneous structure, and there was an interface between the two materials, creating an interface effect in the photocatalytic reaction process. To further study the microstructure of the interface, we conducted a TEM analysis of the samples annealed at 450, 550, 650, and 750 °C, as shown in Figure 3.

Figure 3 shows the low- and high-magnification TEM images of Zn/Ni-PR annealed at different temperatures. As shown in Figure 3a, the nanocomposites were heavily agglomerated at an annealing temperature of 450 °C, with nanoparticle sizes of approximately 20–30 nm. Small nanoparticles of approximately 5 nm were attached to the surfaces of the large particles. The high-resolution TEM images revealed four distinct crystal plane spacings of 0.2812, 0.2808, 0.2005, and 0.2409 nm. The first two lattice fringes corresponded to the (100) crystal plane of the hexagonal wurtzite ZnO, while the latter two crystal plane spacings corresponded to the (200) and (111) crystal planes of cubic-structured NiO. With an increase in the annealing temperature to 550 °C, Figure 3b,b-1 show that the particle size increased to 100 nm and the sample dispersion improved. Furthermore, small particles remained attached to larger particles. The crystal plane spacing of the large particles was 0.2485 nm, which corresponded to the (101) crystal plane of the hexagonal wurtzite ZnO, while the crystal plane spacing of the small particles was 0.1476 nm, corresponding to the (200) crystal plane of the cubic NiO. When the temperature increased above 650 °C, the particles became larger and smaller particles remained attached to them. 

**Figure 4:**
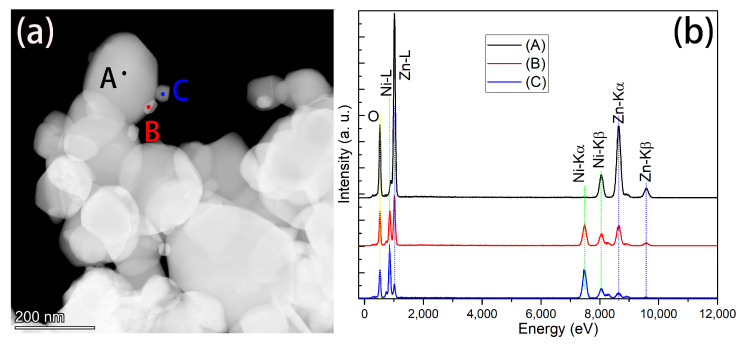
(**a**) HADDF image of the Zn/Ni-550 sample (**b**) EDS spectra corresponding to the three positions shown in (**a**).

Energy dispersive X-ray spectrometer (EDS) mapping was conducted for the Zn/Ni-550 sample, and a high-angle annular dark field (HADDF) image is shown in Figure 3e. According to the EDS mapping of Zn, Ni, and O, the large particles contained Zn and O, while the small particles comprised Ni and O. This was also confirmed by the EDS scan of the points shown in Figure 4. Figure 4a shows the morphology of the Zn/Ni-550 sample. Three positions were marked in the figure: A, B, and C. Position A corresponded to a large particle, Position B corresponded to both large and small particles, and Position C corresponded to a small particle. The EDS spectra collected at these three positions are shown in Figure 4b. Position A primarily contained Zn and O, Position B contained Zn, Ni and O; Position C mainly contained Ni and O. These results confirmed that annealing can change the particle size and degree of crystallization in the precursor. In addition, the annealed samples contained hexagonal wurtzite ZnO and cubic NiO, and large ZnO particles were attached to small NiO particles. The two materials had an obvious heterojunction interface.

**Figure 5:**
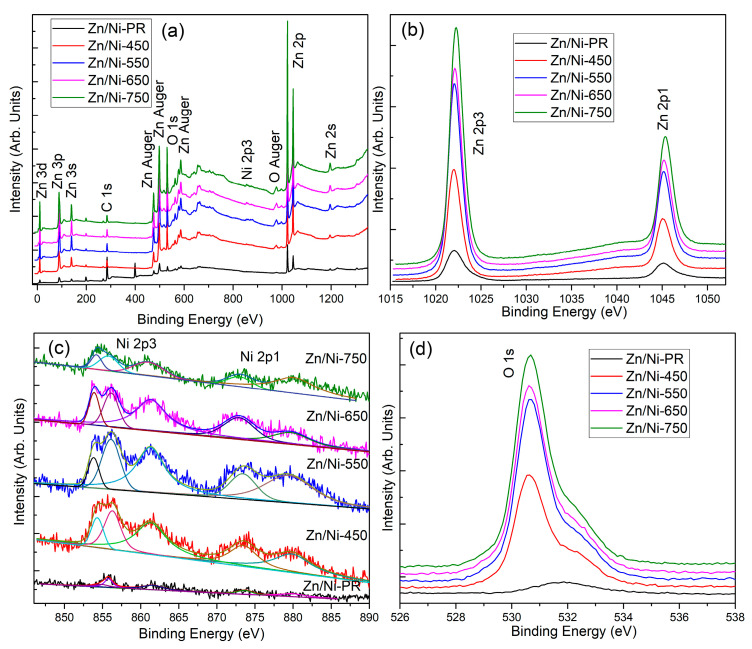
(**a**) XPS spectrum, (**b**) Zn 2p core-level spectrum, (**c**) Ni 2p core-level spectrum, and (**d**) O 1s core-level spectrum of the Zn/Ni-PR and Zn/Ni-*x* sample obtained from different annealing temperatures.

The chemical compositions and valence states of the elements in the samples were studied using XPS. Figure 5 shows both the full and high-resolution XPS spectra for each element in the Zn/Ni-PR samples annealed at different temperatures. As expected, the fully scanned spectra indicated that Zn, O, Ni, and C were present in all samples, and no peaks of other elements were observed. Here, C can be attributed to an adventitious carbon-based contaminant; the binding energy of the C 1s peak at 285.0 eV was used as the reference for calibration. Therefore, all samples were composed of three elements, Zn, Ni, and O), which is consistent with the results of the XRD and EDS analyses. In addition, the peak intensity in the XPS spectrum of Zn/Ni-PR was the weakest, indicating that the Zn/Ni-PR sample contained more organic compounds. With an increase in the annealing temperature, the organic components gradually decomposed and the peaks of the corresponding elements became stronger. To investigate the valence states of the elements, the Zn 2p, Ni 2p, and O 1s high-resolution XPS spectra of all the samples can be analyzed in Figure 5b–d. In Figure 5b, the two peaks at binding energies of 1022.1 and 1045.2 eV can be attributed to Zn 2p_3/2_ and Zn 2p_1/2_ [35], respectively, and the observed spin-orbit splitting of Zn 2p was approximately 23 eV, indicating a normal state of Zn^2+^ in all samples [12]. In addition, the Zn 2p peaks of the annealed sample shifted toward higher binding energies. Figure 5c shows the Ni 2p high-resolution XPS spectra for each sample. The Ni 2p peak of Zn/Ni-PR was weak and could be deconvoluted into five peaks: 854.8, 856.3, 861.5, 873.6, and 879.7 eV. The binding energies at 854.8, 856.3, and 861.5 eV were attributed to the Ni 2p_3/2_ peaks, and the binding energies at 873.6 and 879.7 eV were attributed to the Ni 2p_1/2_ peaks. The Ni 2p _3/2_ peaks were assigned to Ni^2+^ ions in NiO [36,37]. Additionally, the Ni 2p peaks gradually shifted to lower binding energies. The shifts in the binding energies may have occurred because of the different electronegativities of the metal ions and strong interactions between the nanocrystals [38]. The electronegativity of the Ni ions was higher than that of the Zn ions, which caused electrons to be pumped by the Ni ions. In addition, the TEM images clearly show that small NiO nanoparticles were attached to the large ZnO nanoparticles, and this generated the *p-n* heterojunction. Thus, electrons could be transported from the *n*-type ZnO to the *p*-type NiO until the system reached equilibration. Interfacial effects can induce strong interactions. Therefore, the Zn 2p peaks shifted to higher binding energies, whereas the Ni 2p peaks shifted to lower binding energies. Furthermore, Figure 5d shows that the O 1s peak also shifted to a low binding energy. After annealing, the peak intensity of O 1s was stronger than that of Zn/Ni-PR, indicating that Zn and Ni were the main metals in Zn/Ni-PR. Moreover, the organic framework structure decomposed at high temperatures, and the metals, Zn and Ni, reacted with the oxygen in the air and formed ZnO and NiO, respectively. These results suggest that *n*-type ZnO/*p*-type NiO heterojunctions were formed in the nanocomposites.

**Figure 6:**
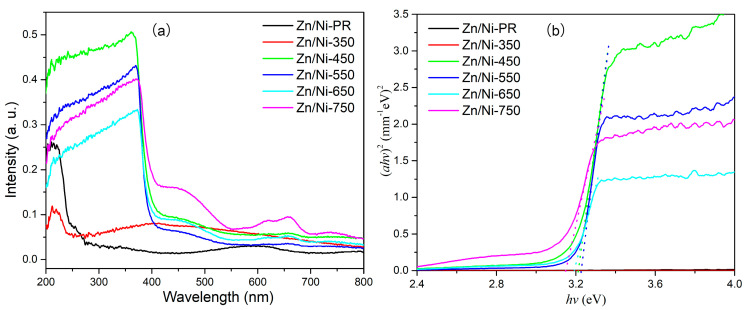
(**a**) UV-vis absorption spectra and (**b**) plot of (*αhv*)^2^ vs. *hv* for the Zn/Ni-PR and Zn/Ni-x samples annealed at the indicated temperatures.

The band gap of a photocatalytic material determines the wavelength range over which the material absorbs radiation. Therefore, the UV-vis absorption spectrum was measured, as shown in Figure 6. For the Zn/Ni-PR and Zn/Ni-350 samples, there was no clear absorption edge, implying that the precursor did not crystallize to form a metal oxide at the corresponding annealing temperature. In contrast, when the annealing temperature was higher than 450 °C, the sample exhibited a clear absorption edge at ~390 nm. The optical bandgap of the ZnO/NiO nanocomposite was calculated using the Tauc formula [39], which is expressed as:

(1) $$\alpha hv=A{\left(hv-E_{g}\right)}^{n}$$

where *v*, A, *h*, *α*, *n*, and E_g_ are the incident light frequency, a constant, Planck’s constant, absorption coefficient, *n* = 1/2 for ZnO and NiO, and optical band gap, respectively. The intersection between the linear extrapolation of the absorption edge and the *x*-axis represents the value of the optical band gap, as shown in Figure 6b. The E_g_ values for Zn/Ni-450, Zn/Ni-550, Zn/Ni-650, and Zn/Ni-750 were 3.21, 3.23, 3.19, and 3.15, respectively. Therefore, with increasing annealing temperature, the optical band gap first increased and then decreased. The increase in the optical band gap was primarily due to the gradual improvement in the crystal quality, while the decrease in the optical band gap was mainly due to the size growth of nanoparticles in the composite owing to the high annealing temperature. In addition, the band gap obtained corresponded to that of the ZnO, but the band gap of the NiO was not detected, which may be due to the small number of NiO particles or a strong ZnO signal overwhelming a weak NiO signal.

**Figure 7:**
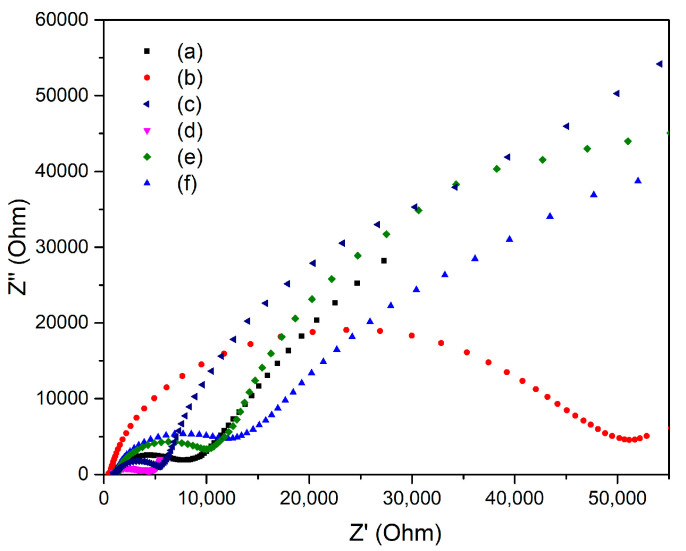
EIS Nyquist plots of (a) commercial ZnO nanoparticles, (b) commercial NiO nanoparticles, (c) Zn/Ni-450, (d) Zn/Ni-550, (e) Zn/Ni-650, and (f) Zn/Ni-750.

To confirm the efficiency of direct electron transfer and separation of photogenerated electrons, EIS Nyquist figure was provided, which is illustrated in Figure 7. In order to compare the EIS spectra of pure ZnO and NiO, commercial ZnO and NiO nanoparticles were used as references. As can be seen by XRD, Zn/Ni-PR, Zn/Ni-250, and Zn/Ni-350, samples did not crystallize to form ZnO/NiO nanocomposites; therefore, data are not presented here. In the EIS spectra, the radius of the arc determines the resistance of the interfacial layer that occurred at the surface of electrode. The small arc radius implies rapid electron transfer, excellent separation efficiency of photogenerated carriers, and a faster interfacial charge transfer process. From the results of our experiment, it can be seen that the radius of the arc of Zn/Ni-550 sample is the smallest. This means that the sample has excellent charge transfer ability and excellent separation ability of photogenerated electron-hole pairs, suggesting that *pn* heterojunctions significantly enhance the interfacial charge transfer rate.

**Figure 8:**
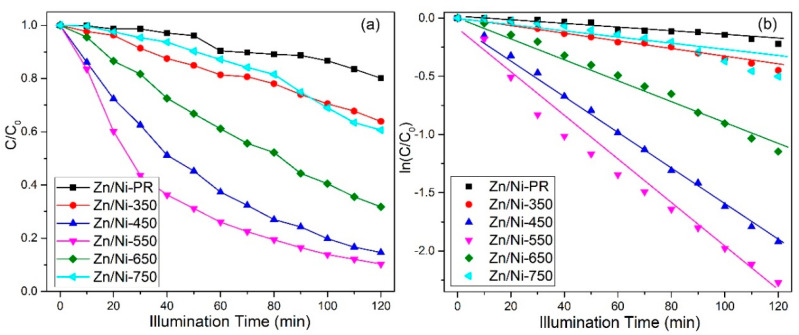
(**a**) Photodegradation of RhB catalyzed by ZnO/NiO nanocomposites irradiated for different times and (**b**) kinetic fits to the experimental data.

To evaluate the photocatalytic activity of the prepared ZnO/NiO nanocomposites, RhB was used as a standard pollutant model for photodegradation experiments. The UV absorption spectrum was used to quantify the concentration of RhB in the solution after irradiation with a certain amount of light (data not shown). The photodegradation efficiency of the RhB was calculated as follows [40]:

Photodegradation efficiency (%) = (1 − C/C_o_) × 100%, (2)

where C_o_ and C represent the absorption coefficients at the absorption equilibrium and immediately after irradiation, respectively. Compared with other metal oxide nanomaterials [41,42,43,44], the degradation efficiency of RhB by Zn/Ni-550 nanomaterials prepared in this paper was improved. Figure 8 shows the variation in C/C_o_ as a function of time. The Zn/Ni-PR and Zn/Ni-350 samples exhibited poor photodegradation performance for RhB organic dyes. This is likely because of the insufficient reaction of metal ions and oxygen during low-temperature annealing and incomplete decomposition of the organic framework. With an increase in the annealing temperature, the degradation efficiency gradually increased, and the best photodegradation performance was achieved at an annealing temperature of 550 °C. However, when the annealing temperature was higher than 550 °C, the photodegradation efficiency decreased. The experimental data shown in Figure 8a were fitted linearly using the Equation [12]:

(3) $$\mathrm{In}(\frac{C}{C_{0}})=-kt$$

where *k* and *t* are the photodegradation rate constant and reaction time, respectively. Figure 8b shows a plot of $\mathrm{In}(\frac{C}{C_{0}})$ versus the illumination time. This relationship is approximately linear, implying that the photodegradation process conformed to pseudo-first-order kinetics. According to the linear fit, the *k* values for photodegradation of RhB by the Zn/Ni-PR, Zn/Ni-350, Zn/Ni-450, Zn/Ni-550, Zn/Ni-650, and Zn/Ni-750 samples were 0.00148, 0.0033, 0.0153, 0.019, 0.0090, and 0.00262, respectively. The Zn/Ni-550 sample exhibited the highest photocatalytic activity. The characterization methods used in this study, such as XRD, TEM and XPS, confirmed that the organic framework was completely decomposed, and a heterojunction interface was formed between the ZnO and NiO at an annealing temperature of 550 °C. The ZnO/NiO nanocomposites also had suitable particle sizes. At low annealing temperatures, ZnO and NiO did not crystallize and the organic framework was not completely decomposed. At temperatures above 550 °C, the size of the ZnO and NiO particles increased sharply in the order of microns, the quantum effect of the nanomaterials was eliminated, and the decrease in specific surface area resulted in a less potent reaction between the material and dye.

**Scheme 2:**
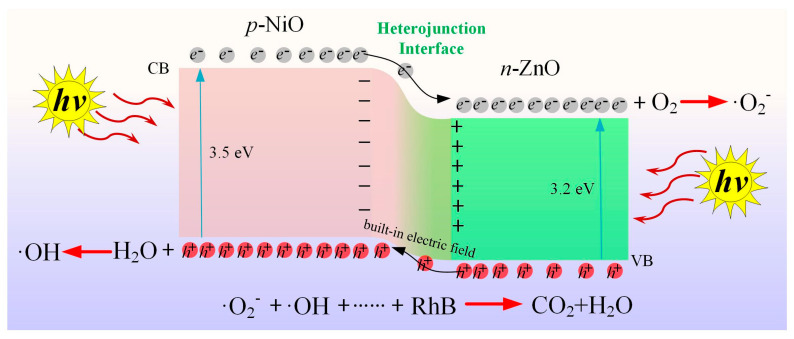
Schematic diagram of RhB degradation mechanism of ZnO/NiO nanocomposite under light irradiation.

Based on the abovementioned experimental results and analysis, the mechanism by which the ZnO/NiO nanocomposite photocatalytically degraded the organic dye is proposed. The schematic diagram of RhB degradation mechanism of ZnO/NiO nanocomposited under UV light irradiation is shown in Scheme 2. When the *p*-type NiO and the *n*-type ZnO formed a *pn* heterojunction at a suitable annealing temperature, there was a built-in electric field at the interface of the heterojunction. Owing to the presence of this built-in electric field, the inner electric field caused the *p*-type NiO region to have a negative charge. On the contrary, the *n*-type ZnO region had a positive charge to achieve charge balance. When the ZnO/NiO nanocomposite was irradiated with UV light, the electrons in the valence band were excited up to the conduction band to form photogenerated electrons, and photogenerated holes were generated in the valence band. The photogenerated electrons were transferred from the conduction band of the *p*-type NiO to that of *n*-type ZnO due to the inner electric field. The electrons reacted with the oxygen molecules adsorbed in the dye to form ∙O_2_^−^ [45]. Simultaneously, holes were transported from the valence band of the *n*-type ZnO to that of the *p*-type NiO, and the holes interacted with the water in the dye to form ∙OH [46]. Therefore, the recombination of photogenerated electrons and holes is largely inhibited. In addition, the ∙O_2_^−^ and ∙OH radicals functioned together to degrade the dye into non-polluting products, such as carbon dioxide and water. In summary, the heterojunction between the *n*-type ZnO and the *p*-type NiO play a very important role in impeding the recombination of the photogenerated electron-hole pairs and improving the photocatalytic efficiency.

## 4. Conclusions

In this study, a simple low-cost coprecipitation method was used to prepare a Zn and Ni duplex metal–organic framework structure, Zn/Ni-PR, which was annealed at different temperatures to form nanocomposites. The crystal structure, morphology, and optical properties of the nanocomposites were investigated using XRD, SEM, TEM, XPS, and UV-vis absorption spectroscopy. The results showed that ZnO/NiO nanocomposites composed of hexagonal wurtzite ZnO and cubic NiO were formed after Zn/Ni-PR was annealed. ZnO was the main component in the nanocomposites. TEM and EDS mapping revealed that large ZnO particles were attached to small NiO nanoparticles, and a clear heterojunction boundary was formed between the two materials. The results of the photocatalytic degradation indicated that ZnO and NiO did not crystallize, and the organic framework structure was not completely decomposed at low annealing temperatures. On the contrary, when the temperature was above 550 °C, the increased particle size of ZnO and NiO led to a decrease in the specific surface area. The excellent photodegradation activity of the Zn/Ni-550 sample was ascribed to the outstanding quality of *pn* heterojunctions. The heterojunction between the *n*-type ZnO and the *p*-type NiO plays an important role in impeding the recombination of photogenerated electron-hole pairs and improving the photocatalytic efficiency.

## Data Availability

No applicable.
